# The long non-coding RNA *LINC00152* is essential for cell cycle progression through mitosis in HeLa cells

**DOI:** 10.1038/s41598-017-02357-0

**Published:** 2017-05-23

**Authors:** Linda Nötzold, Lukas Frank, Minakshi Gandhi, Maria Polycarpou-Schwarz, Matthias Groß, Manuel Gunkel, Nina Beil, Holger Erfle, Nathalie Harder, Karl Rohr, Jakob Trendel, Jeroen Krijgsveld, Thomas Longerich, Peter Schirmacher, Michael Boutros, Sylvia Erhardt, Sven Diederichs

**Affiliations:** 1 Division of RNA Biology & Cancer, German Cancer Research Center (DKFZ), 69120 Heidelberg, Germany; 2 Center for Molecular Biology (ZMBH), DKFZ-ZMBH Alliance and CellNetworks Excellence Cluster, Heidelberg University, 69120 Heidelberg, Germany; 3 Hartmut Hoffmann-Berling International Graduate School of Molecular and Cellular Biology (HBIGS), Heidelberg University, 69129 Heidelberg, Germany; 4 ViroQuant-CellNetworks RNAi Screening Facility, BioQuant Center, Heidelberg University, 69120 Heidelberg, Germany; 5 Department of Bioinformatics and Functional Genomics, Biomedical Computer Vision Group, Heidelberg University, BioQuant, IPMB, and German Cancer Research Center (DKFZ), 69120 Heidelberg, Germany; 6 German Cancer Research Center (DKFZ), Excellence Cluster CellNetworks, Heidelberg University, 69120 Heidelberg, Germany; 7 European Molecular Biology Laboratory (EMBL), Genome Biology Unit, 69117 Heidelberg, Germany; 8 Institute of Pathology University Hospital RWTH Aachen, 52074 Aachen, Germany; 9 Institute of Pathology, University Hospital Heidelberg, 69120 Heidelberg, Germany; 10 Division of Signaling and Functional Genomics, German Cancer Research Center (DKFZ) and Department of Cell and Molecular Biology, Medical Faculty Mannheim, Heidelberg University, 69120 Heidelberg, Germany; 11 Division of Cancer Research, Dept. of Thoracic Surgery, Medical Center - University of Freiburg, 79106 Freiburg, Germany; 12 Faculty of Medicine, University of Freiburg, 79085 Freiburg, Germany; 13 German Cancer Consortium (DKTK), 79104 Freiburg, Germany; 14 Definiens AG, 80636 Munich, Germany

## Abstract

In recent years, long non-coding RNA (lncRNA) research has identified essential roles of these transcripts in virtually all physiological cellular processes including tumorigenesis, but their functions and molecular mechanisms are poorly understood. In this study, we performed a high-throughput siRNA screen targeting 638 lncRNAs deregulated in cancer entities to analyse their impact on cell division by using time-lapse microscopy. We identified 26 lncRNAs affecting cell morphology and cell cycle including *LINC00152*. This transcript was ubiquitously expressed in many human cell lines and its RNA levels were significantly upregulated in lung, liver and breast cancer tissues. A comprehensive sequence analysis of *LINC00152* revealed a highly similar paralog annotated as *MIR4435-2HG* and several splice variants of both transcripts. The shortest and most abundant isoform preferentially localized to the cytoplasm. Cells depleted of *LINC00152* arrested in prometaphase of mitosis and showed reduced cell viability. In RNA affinity purification (RAP) studies, *LINC00152* interacted with a network of proteins that were associated with M phase of the cell cycle. In summary, we provide new insights into the properties and biological function of *LINC00152* suggesting that this transcript is crucial for cell cycle progression through mitosis and thus, could act as a non-coding oncogene.

## Introduction

Long non-coding RNAs (lncRNAs) constitute a heterogeneous group commonly defined as transcripts of more than 200 nucleotides that lack an extended open reading frame (ORF)^1^. In recent years, studies have linked lncRNAs to a wide variety of physiological and pathological mechanisms, including cell cycle^2^ and cancer development^3^. Several lncRNAs, e.g. *gadd7* ^4^ and *lncRNA-RoR* ^5^, modulate cell cycle regulators such as cyclins, cyclin-dependent kinases (CDKs), CDK inhibitors and p53 and thus, provide an additional layer of flexibility and robustness to cell cycle progression^2^. In addition, some lncRNAs are linked to mitotic processes such as centromeric satellite RNA, which is essential for kinetochore formation and thus crucial for chromosome segregation during mitosis in humans^6^ and flies^7^. Another nuclear lncRNA, *MA-linc1*, regulates M phase exit by functioning *in cis* to repress the expression of its neighbouring gene *Purα*, a regulator of cell proliferation^8^. Since deregulation of the cell cycle is closely associated with cancer development and growth, cell cycle regulatory lncRNAs may have oncogenic properties.

To date, gene silencing by RNA interference (RNAi) enabled high-throughput screens in cells and organisms of different species up to a genome-wide level^9^. The generated loss-of-function phenotypes have made it possible to identify new genes that are involved in various pathways and cellular processes including cell division^10, 11^, signal transduction^12^ and cancer^13^. RNAi screens in human material are cell-based and enable the detection of a diverse set of phenotypes by a variety of assays ranging from simple cell viability readouts to high-content assays using time-lapse microscopy^14^. Notably, siRNA screens in human cells^10, 11^ and Drosophila^15, 16^ were only used to analyse novel protein-coding genes affecting cell division. These investigations revealed, for example, proteins required for centromere propagation (*CAL1*, *CENP-C*, *CYCA*, *RCA1*)^15, 16^, spindle assembly (*PTGER2*, *ECT2*, *CABP7*, *C13orf23*) and cytokinesis (*AURKB*, *INCENP*, *TOR1AIP1*)^11^. However, RNAi may also provide a valuable approach to gain insights into the biological functions of novel lncRNAs^17^. In the past years, siRNA libraries targeting lncRNAs have been generated but only few functional lncRNAs were classified. One siRNA library consisted of three siRNAs per candidate targeting 286 putative lncRNAs^18^ present in the full-length long Japan collection of sequenced human full length cDNA^19^. To identify lncRNAs required for cell proliferation, this library was screened using Bromodeoxyuridine (BrdU) incorporation assays. Another study used an endoribonuclease-prepared siRNA (esiRNA) library designed to target 1,779 individual human lncRNAs^20^. esiRNAs are pools of several hundreds of individual siRNAs generated by enzymatic digestion of a typically 300- to 600-base-pairs-long dsRNA derived from a single target transcript^21^. This technology has also been successfully used for targeting mouse lncRNAs in a loss-of-function screen detecting genes implicated in the maintenance of pluripotency in embryonal stem cells^22^. However, the esiRNA screen targeting human lncRNAs demonstrated only a proof-of-principle for this tool and did not focus on the evaluation of any phenotype.

The profound investigation of lncRNAs and their regulatory roles is essential as it may help to comprehensively understand complex physiological and pathological processes including cell cycle and cancer. Hence, we aimed to identify lncRNAs as novel regulators of cell cycle progression. In the present study, we performed a customized focused RNAi screen targeting 638 lncRNAs deregulated in tumours. Time-lapse microscopy revealed 26 putative lncRNAs affecting cell morphology and cell cycle. Furthermore, we characterized the properties and biological function of lncRNA *LINC00152* which was linked to mitosis. These endeavours highlight the role of lncRNAs in cell division.

## Results

### Time-lapse microscopy RNAi screen identified lncRNAs affecting cell division

**Figure 1:**
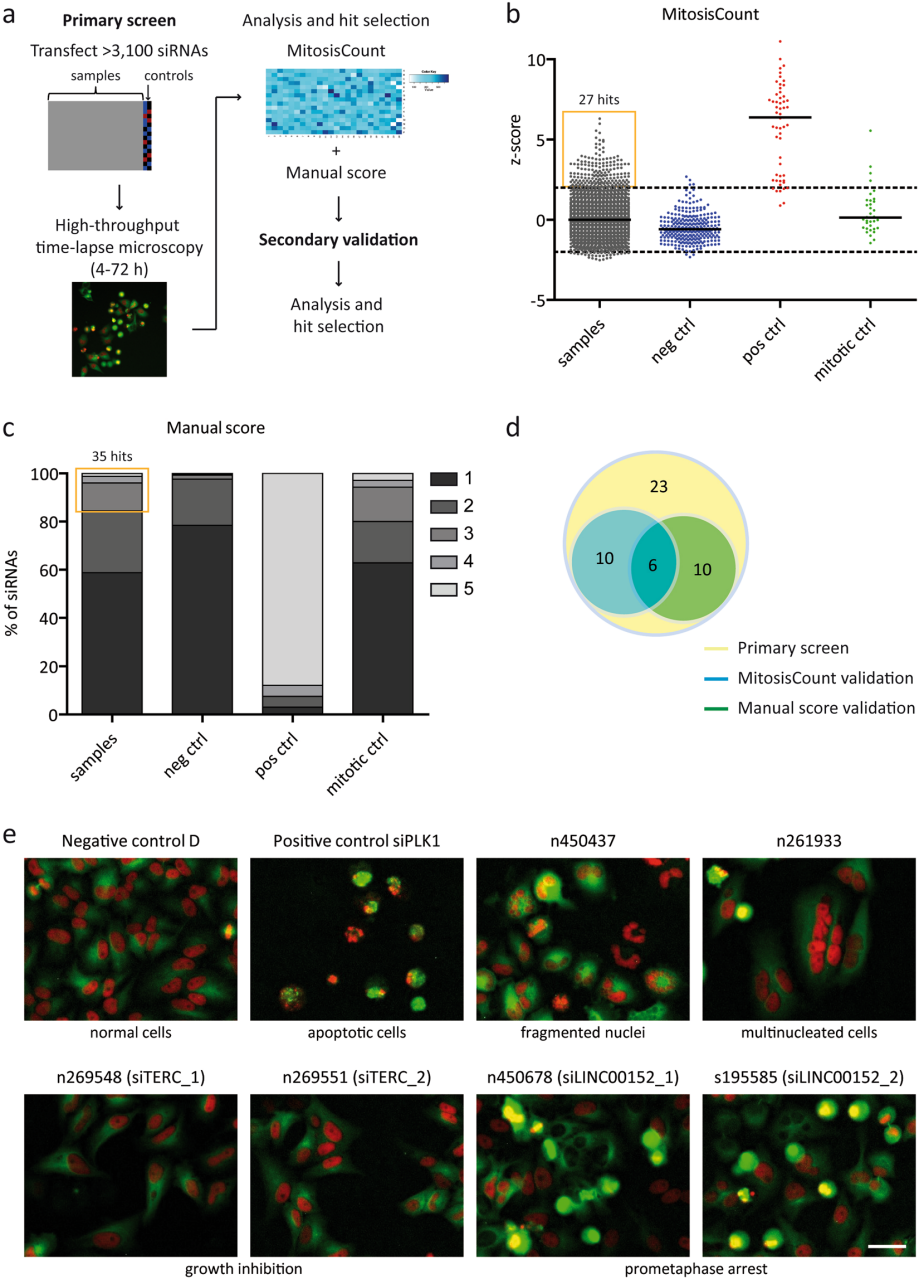
High-throughput RNA interference screen for cell cycle regulators. (**a**) Schematic overview of the siRNA screening strategy. (**b**) Bioinformatical analysis was performed by calculating the z-scores for MitosisCount. Black lines represent the median of z-scores. Dashed lines indicate the cut-off for potential phenotypes. (**c**) Manual analysis was accomplished by assigning a manual score representing the strength of any kind of aberrant phenotype. 1 = normal; 5 = strong phenotype. (**d**) Venn diagram of hits from the primary screen and secondary validation. (**e**) Image sections of exemplary phenotypes discovered by time-lapse microscopy (red: histone H2B, green: tubulin). The siRNA IDs are stated above the images, phenotypes are described below the images. Images were acquired at 10x magnification. Scale bar represents 100 μm.

A comprehensive expression map of over 17,000 ncRNAs in three major cancer entities and normal tissues was previously generated in our lab using the NCode Human Non-coding RNA Microarray from Life Technologies (Polycarpou-Schwarz M. *et al*., in preparation; Roth A. *et al*., in preparation). The comparison of primary human lung, breast and liver cancer with normal tissue from the respective organs in a large set of primary patient samples (N = 150) identified hundreds of non-coding transcripts specifically expressed or silenced in human cancer. More precisely, 638 ncRNAs were identified as significantly upregulated in at least one of the tumor entities with a minimum fold change FC ≥ 2.0 and a maximum corrected p-value p < 0.05. Based on this analysis, an siRNA library was designed targeting these 638 lncRNAs upregulated in lung, liver or breast cancer with up to five individual siRNAs per target (Supplementary Table S1). With these siRNAs, we analysed the impact of potentially oncogenic lncRNAs on cell morphology and cell cycle progression in a high-throughput RNAi screen. To monitor cell division phenotypes, we used time-lapse microscopy to image HeLa cells stably expressing core histone 2B tagged with mCherry (H2B-mCherry) and α-tubulin tagged with green fluorescent protein (GFP-α-tubulin)^11^. A schematic of the screening strategy is presented in Fig. 1a.

The data was evaluated bioinformatically by using the total number of mitotic events (MitosisCount) to calculate the z-scores (Fig. 1b). Approximately 90% of the positive cell viability controls targeting *COPB2*, *KIF11* and *PLK1* had a z-score ≥2 for MitosisCount. In turn, more than 95% of the negative controls, which included three independent non-targeting siRNA controls and wells without siRNAs, had a z-score <2. However, only a minority of the positive controls for mitotic defects (*CENPA*, *ZW10*, *HJURP*) reached a z-score ≥2 for MitosisCount. That means that either the siRNAs were inefficient in targeting those genes or the bioinformatical readout was not sensitive enough to detect those more subtle phenotypes. Therefore, the data was also evaluated by manual inspection of the images (Fig. 1c).

The primary screen identified 27 and 35 lncRNAs with at least two independent siRNAs affecting MitosisCount and manual score, respectively (Fig. 1b,c and Supplementary Tables S1,S2). The overlap between both analyses comprised 13 lncRNAs yielding in total 49 potential hits. To reduce potential off-target candidates, we performed a validation screen revealing in total 26 lncRNAs affecting cell morphology or cell cycle progression (Fig. 1d and Supplementary Table S2). Among those, 16 candidates were confirmed with each the computational and the manual approach including six lncRNAs found in both analyses. The phenotypes that were discovered during the siRNA screen included reduced cell proliferation, mitotic aberrations, abnormal nuclear shapes and numbers as well as apoptosis (Fig. 1e). The two lncRNA hits with the highest expression and most efficient knockdown were *TERC* and *LINC00152*. The knockdown of *TERC*, the well-studied telomerase RNA component^23^, was already associated with a rapid, telomerase-independent growth arrest^24^. *LINC00152* knockdown caused cells to arrest in prometaphase of mitosis - a phenotype, which had not been studied previously. Since the structure and function of this lncRNA were overall poorly characterized, we selected it for in-depth analysis.

### *LINC00152* and its paralog *MIR4435-2HG* give rise to several splice variants

**Figure 2:**
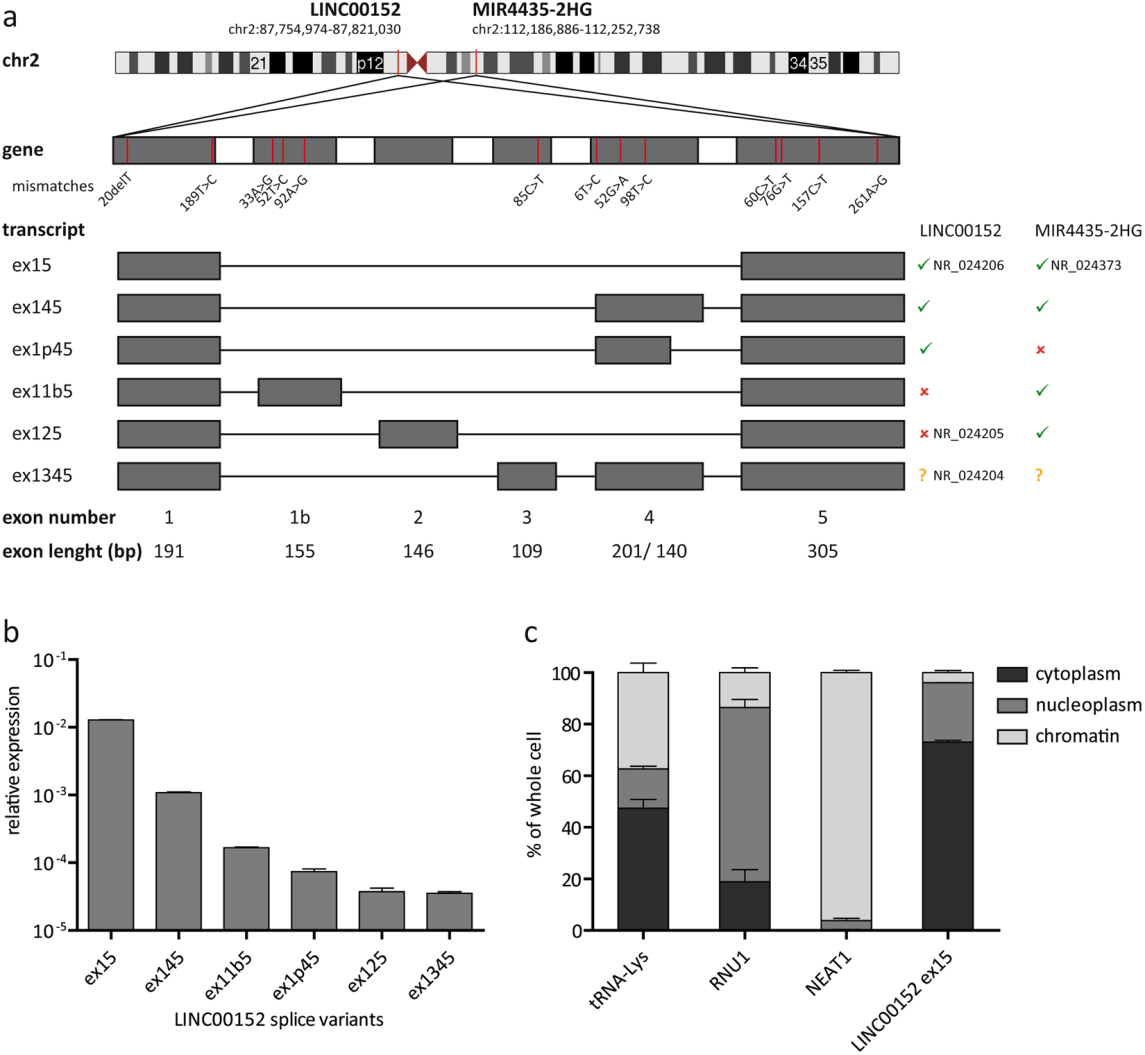
*LINC00152* transcripts and their subcellular localization. (**a**) The exonic regions of *LINC00152* and *MIR4435-2HG* differed only by 13 base pairs (upper panel, mismatch numbers were counted for each exon separately). Exons were numbered according to their genomic order and transcripts were named according to their comprised exons. Both paralogs were transcribed into several isoforms (lower panel) and had splice variants ex15 and ex145 in common (right panel). (**b**) Relative expression of all identified *LINC00152* splice variants determined by RT-qPCR normalized to Cyclophilin A expression. Note that the graph is depicted in Log10 scale. (**c**) Subcellular localization of *LINC00152* was determined using cell fractionation. After separation of cytoplasm, nucleoplasm and chromatin, RNA was extracted from all fractions and *LINC00152* expression was measured by RT-qPCR. tRNA-Lys, RNU1 and NEAT1 were used as cytoplasmic, nucleoplasmic and chromatin marker, respectively. N = 3. Error bars indicate SEM.

Three splice variants of *LINC00152* (NR_024204, NR_024205 and NR_024206) were already annotated in the Human Genome hg19 assembly in the reference sequence database RefSeq and mapped to the short arm of chromosome 2p11.2 (Fig. 2a). To confirm the existence of these transcripts and their full-length sequence, we performed PCR as well as 3′ and 5′ rapid amplification of cDNA ends (RACE) experiments. Surprisingly, this led to the detection of additional isoforms including an actively expressed *LINC00152* paralog, annotated as *MIR4435-2HG* (RefSeq: NR_024373) on chromosome 2q13 (Fig. 2a). *MIR4435-2HG* differed from *LINC00152* only by 13 exonic single nucleotide exchanges, which prevented a discrimination of the transcripts by RT-qPCR (Supplementary Fig. S1). All splice variants contained the first and the last exon with similar 5′ and 3′ ends (Supplementary Fig. S2). However, only splice variants ex15 and ex145 were detected from both loci whereas all other isoforms seemed to be specific for either *LINC00152* or *MIR4435-2HG* (Fig. 2a, right panel).

To elucidate the expression level of the identified transcript isoforms, primers specifically amplifying each splice variant were used for RT-qPCR (Fig. 2b). The shortest splice variant containing the first and the last exon (ex15) was the by far most abundant transcript. Its relative expression was 10-fold and 100-fold higher compared to the second most abundant transcript ex145 and all other isoforms, respectively. Of note, using the comparative C_T_ method assumed the same amplification efficiency for all amplicons^25^, so that different qPCR amplicons could not necessarily be compared quantitatively. Nevertheless, cloning and sequencing of PCR and RACE fragments supported the RT-qPCR results. The high abundance of splice variants ex15 and ex145 was probably due to the fact that both were transcribed from the *LINC00152* and the *MIR4435-2HG* locus (Fig. 2a, right panel). The annotated isoform ex1345 (NR_024204) was never cloned in full-length from HeLa cDNA but could be detected by RT-qPCR (Fig. 2b). Assuming that the most abundant alternatively spliced transcript had the biggest biological relevance, we focused on splice variant ex15 for the following experiments.

Assessing the non-coding properties of *LINC00152* isoforms by several *in silico* tools revealed open reading frames^26^ smaller than 100 codons, Coding Potential Scores^27^ below 0, negative PhyloCSF (codon substitution frequencies) scores^28^ and no experimentally identified peptide in PeptideAtlas^29^. Together, these data strongly indicated that *LINC00152* indeed was a long non-coding RNA (Supplementary Fig. S3).

To gain first insights into its function, the subcellular localization of *LINC00152* ex15 was determined using cell fractionation. Approximately 70% of this *LINC00152* transcript was detected in the cytoplasm of HeLa cells whereas most of the remaining transcripts were found in the nucleoplasm with almost no detection of *LINC00152* in the chromatin fraction (Fig. 2c). Hence, the most abundant isoform of *LINC00152* was mainly cytoplasmic.

### *LINC00152* is upregulated in cancer tissues and expressed in a variety of human cell lines

**Figure 3:**
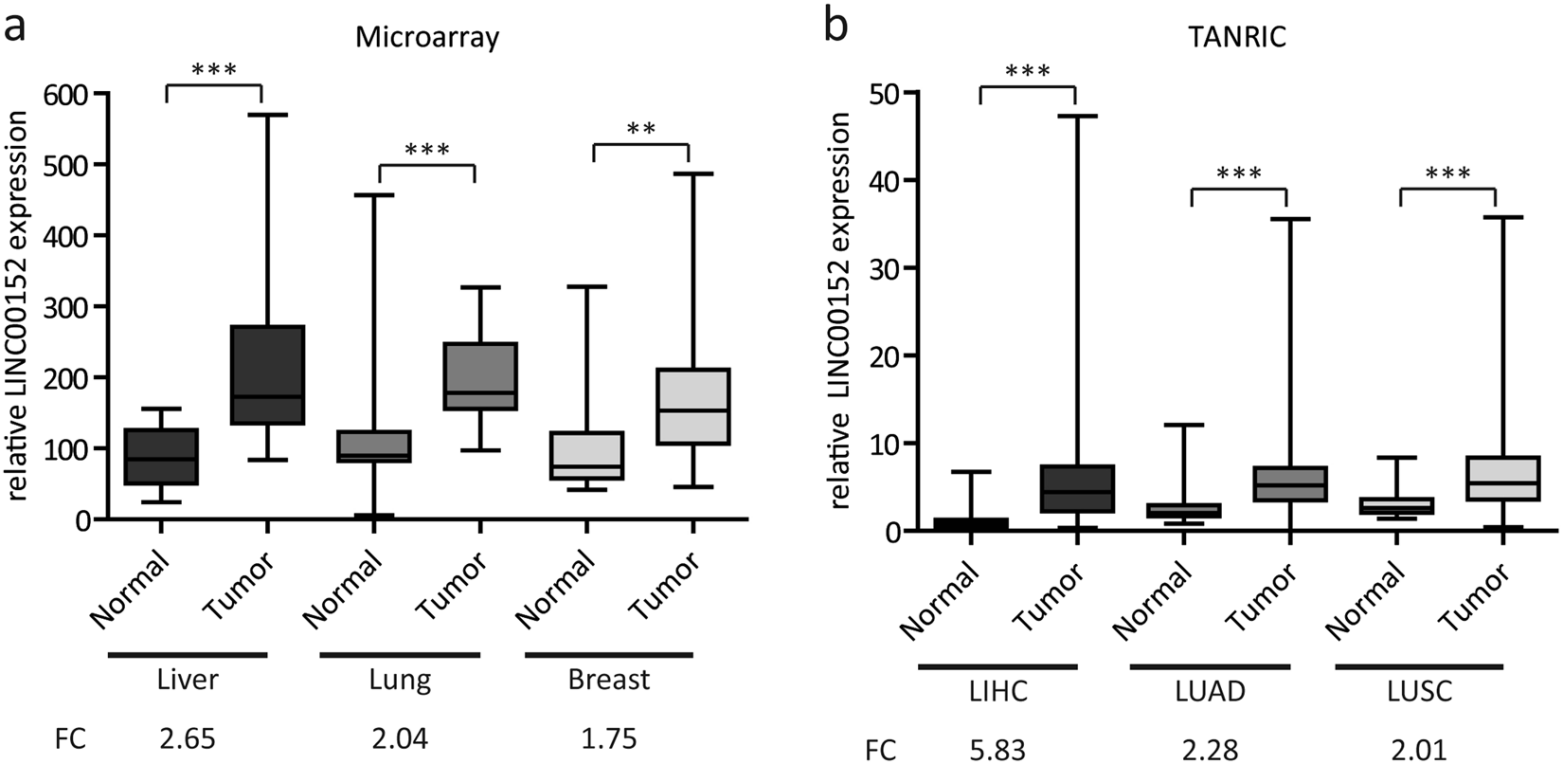
*LINC00152* expression in different human tumour tissues. (**a**) Microarray profiling of the *LINC00152* expression in 150 patient samples (7 normal liver vs. 32 liver cancer, 27 normal lung vs. 27 matched lung cancer, 13 normal breast vs. 44 breast cancer). (**b**) Relative *LINC00152* expression profiles in liver hepatocellular carcinoma (LIHC), lung adenocarcinoma (LUAD) and lung squamous cell carcinoma (LUSC) obtained from The Atlas of non-coding RNA in Cancer (TANRIC)^30^. Boxes show the median, 25^th^ and 75^th^ percentiles, and whiskers range from minimum to maximum. FC = fold change. *p < 0.05; **p < 0.01; ***p < 0.001.

A comprehensive microarray profiling in lung, liver, breast cancer revealed a significant up-regulation of *LINC00152* in liver (2.7-fold), lung (2.0-fold) and breast (1.8-fold) cancer compared to healthy tissue of the respective organs (Fig. 3a). These results were consistent with *LINC00152* expression profiles from the Atlas of ncRNA in Cancer (TANRIC) database^30^. In liver hepatocellular carcinoma (LIHC), lung adenocarcinoma (LUAD) and lung squamous cell carcinoma (LUSC), *LINC00152* transcript levels were also significantly upregulated compared to the corresponding normal tissues (Fig. 3b).

Many lncRNAs show more tissue-specific expression patterns when compared with protein-coding genes^1, 31^. Thus, we examined the relative expression of *LINC00152* in a panel of cancerous and non-malignant cells derived from 17 different tissue entities by RT-qPCR. The results clearly showed that *LINC00152* was ubiquitously detectable with varying expression levels in all human cell lines, except Ramos cells originating from Burkitt′s lymphoma (Supplementary Fig. S4). Taken together, these findings provide evidence for the elevated transcript levels of *LINC00152* in several cancer types and its ubiquitous expression in many human cell lines encouraging further investigation of its potential function in human malignancies.

### *LINC00152* knockdown results in a prometaphase arrest

**Figure 4:**
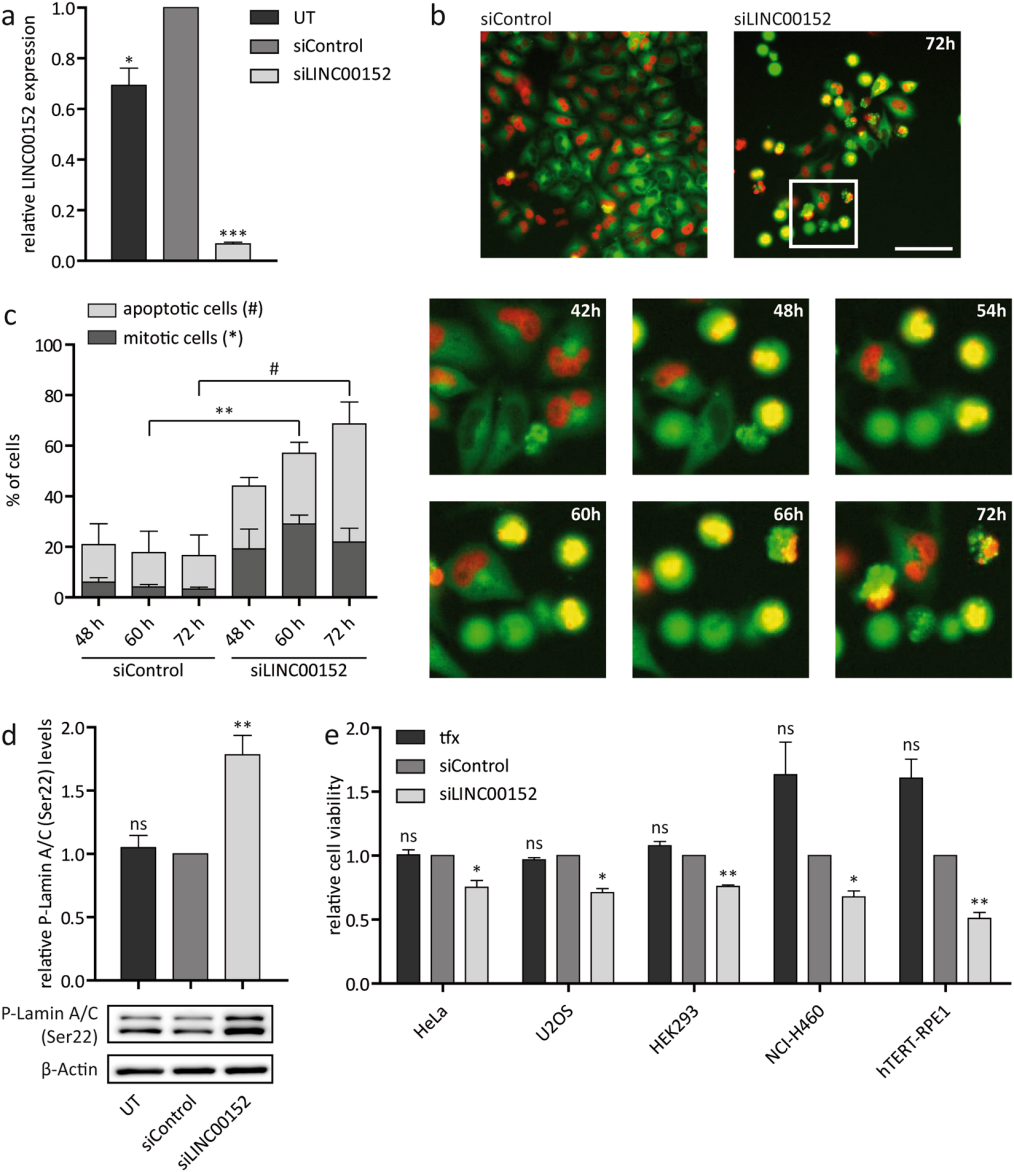
Live-cell microscopy revealed a cell division phenotype with high temporal resolution. (**a**) Relative expression of *LINC00152* detected by RT-qPCR and normalized to *Cyclophilin A* expression in untreated HeLa cells and cells transfected with 30 nM siPOOLs. (**b**) Endpoint images (upper panel) and magnified time-lapse microscopy pictures (lower panel) of HeLa cells treated with 30 nM siPOOLs. Images were acquired at 20x magnification and scale bar represents 200 μm. Live-cell imaging revealed a mitotic arrest in prometaphase and reduced cell viability after *LINC00152* depletion. Time depicts hours after transfection. (**c**) Quantification of mitotic (significance *) and apoptotic (significance ^#^) cells at three different time points after RNAi knockdown. (**d**) Validation of mitotic arrest by quantification of P-Lamin A/C (Ser22) levels detected by Western blot and normalized to β-Actin levels in HeLa cells. (**e**) Relative cell viability detected by CellTiter-Glo Luminescent Cell Viability Assay in different cell lines treated with siPOOLs. UT = untreated; tfx = transfection reagent only. N = 3. Error bars indicate SEM. *^/#^p < 0.05; **p < 0.01; ***p < 0.001; ns = not significant.

In order to study the phenotype upon *LINC00152* knockdown in more detail, we used enzymatically generated pools of siRNA referred to as siPOOLs^32^. These siPOOLs contained 30 siRNAs, each targeting the gene of choice with carefully selected, different seed regions to minimize potential off-target effects. Less than 10% of relative *LINC00152* expression was left in HeLa cells 60 hours post transfection with 30 nM of siLINC00152 (Fig. 4a). Time-lapse microscopy of HeLa cells depleted of *LINC00152* revealed an arrest in mitosis, more precisely in prometaphase, which could last up to ten hours under *LINC00152* knockdown conditions (Fig. 4b). This phenotype appeared strongest 60 hours after siPOOL-mediated knockdown of *LINC00152* (Fig. 4c). In addition, the number of apoptotic cells increased significantly over time (Fig. 4b,c). Phosphorylated lamins A and C (P-Lamin A/C) provide a well-established marker for prometaphase because these proteins are phosphorylated at the onset of mitosis and dephosphorylated after chromosome segregation driving disassembly and assembly of the nuclear envelope, respectively^33^. Thus, the occurrence of cells arrested in prometaphase was confirmed by significantly increased P-Lamin A/C levels (1.8-fold) after *LINC00152* knockdown compared to control cells (Fig. 4d).

Next, we wanted to examine whether *LINC00152* was also important for cell cycle progression in other human cell lines. However, we did not observe a significant increase of P-Lamin A/C levels in other cells 60 hours after *LINC00152* depletion. Nevertheless, cell viability decreased significantly upon *LINC00152* knockdown in the cell lines NCI-H460, HEK293, U2OS and hTERT-RPE1 derived from lung cancer, embryonic kidney, osteosarcoma and retina, respectively (Fig. 4e), indicating a broadly relevant function of *LINC00152* in cell proliferation. Overexpression of the two most abundant *LINC00152* isoforms in HeLa cells did not affect cell proliferation (Supplementary Fig. S5).

**Figure 5:**
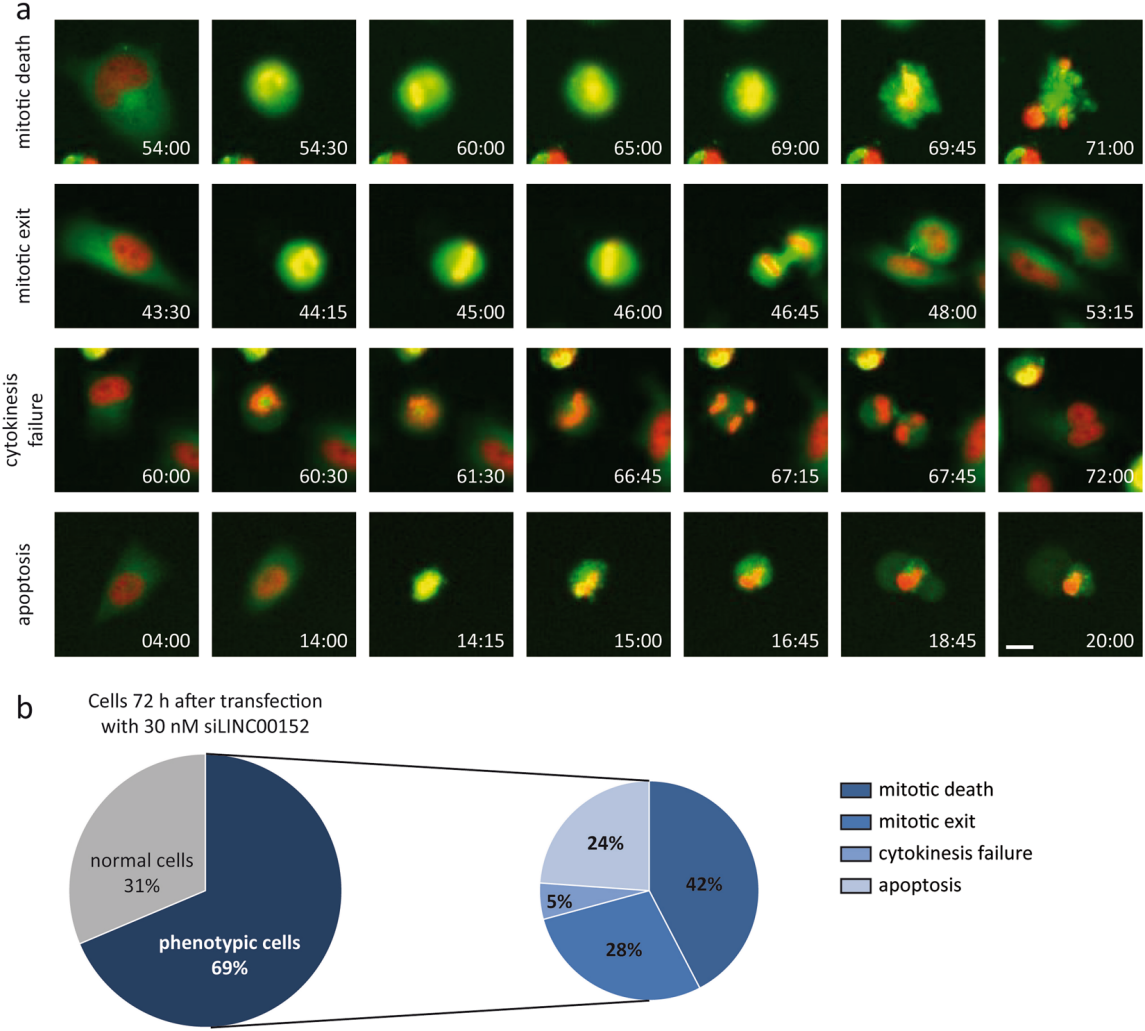
Analysis of cell fates after *LINC00152* depletion. (**a**) Images from movies of HeLa cells after *LINC00152* knockdown by 30 nM siPOOL showed prometaphase arrest followed by mitotic death, mitotic exit or cytokinesis failure. Some cells also underwent apoptosis at an early stage without prior mitotic arrest. Time depicts hours after transfection. Images were acquired at 20x magnification and scale bar represents 20 μm. (**b**) Left circle portrays the effect on cells 72 hours after transfection with siLINC00152 (compare with Fig. 4b). Right circle represents a quantification of the cell fates depicted in (A) occurring in phenotypic cells. N = 3.

To determine whether apoptosis was a primary or secondary consequence of *LINC00152* knockdown, we determined the time-resolved order of events and fates of aberrant cells (Fig. 5a,b). Indeed, three quarters of the phenotypic HeLa cells first exhibited a prometaphase arrest followed by three different fates. Nearly half of the arrested cells (42%) were subject to apoptosis, as evidenced by the fragmentation of the nucleus and formation of apoptotic bodies (Fig. 5a upper panel, b). Alternatively, 28% of defective cells could exit mitosis (Fig. 5a second panel, b) whereas only a small portion (5%) displayed cytokinesis failure (Fig. 5a third panel, b). The remaining quarter of aberrant cells directly underwent cell death without prior mitotic arrest (Fig. 5a lower panel, b). Notably, direct apoptosis occurred relatively early (between 12 to 24 hours) after transfection in *LINC00152*-depleted cells as well as in cells treated with siControl. Therefore, apoptosis was unlikely a direct consequence of *LINC00152* knockdown. In summary, *LINC00152* depletion resulted in a severe prometaphase arrest of several hours mainly followed by apoptosis. These results indicated that *LINC00152* was essential for progression through cell cycle.

Intriguingly, *LINC00152* and *MIR4435-2HG* are located close to genes that are crucial for cell cycle and apoptosis including ANAPC1P1 and ANAPC1^34^, BUB1^35^ and BCL2L11^36^. Nevertheless, the expression levels of these transcripts remained unaltered after *LINC00152* knockdown (Supplementary Fig. S6). Moreover, several recent publications proposed that *LINC00152* regulated the expression of genes involved in cell proliferation and migration pathways including E-Cadherin in the gastric cancer cell lines HGC-27 and SGC-7901^37^, EpCAM in HepG2 and MHCC-97H cells derived from hepatocellular carcinoma^38^, EGFR in the gastric cancer cells MGC803 and HGC-27^39^, p15 and p21 in BGC-823 and SGC-7901 cells derived from gastric cancer^40^, mTOR, GOLPH3, KIF14, PRKCA and SMYD3 in the breast cancer cell line MDA-MB-231^41^, as well as miR-193a-3p and ERBB4 in the colon cancer cell lines SW620 and HT29^42^. However, we did not observe any of the deregulations of these previously reported genes in HeLa cells depleted of *LINC00152* using an siPOOL (Supplementary Fig. S6). Thus, *LINC00152* may be part of several molecular pathways in different cell lines and might be involved in cell cycle regulation by associating with a specific set of proteins, similar to ZFAS1^43^ and GAS5^44^.

### *LINC00152* might interact with a protein network associated with M phase

To reveal the molecular mechanism of *LINC00152*, we investigated RNA-protein interactions *in vivo* by RNA affinity purification (RAP). To identify the protein interactome of the lncRNA, we used two independent sets of biotinylated antisense probes (raPOOLs) that hybridize to *LINC00152* RNA to purify this endogenous RNA and its endogenous associated proteins from cross-linked cell lysates^45–48^. Using mass spectrometry analysis, we found 132 proteins enriched in the *LINC00152* pulldowns compared to two raPOOLs not targeting any human RNA (Supplementary Table S3).

**Figure 6:**
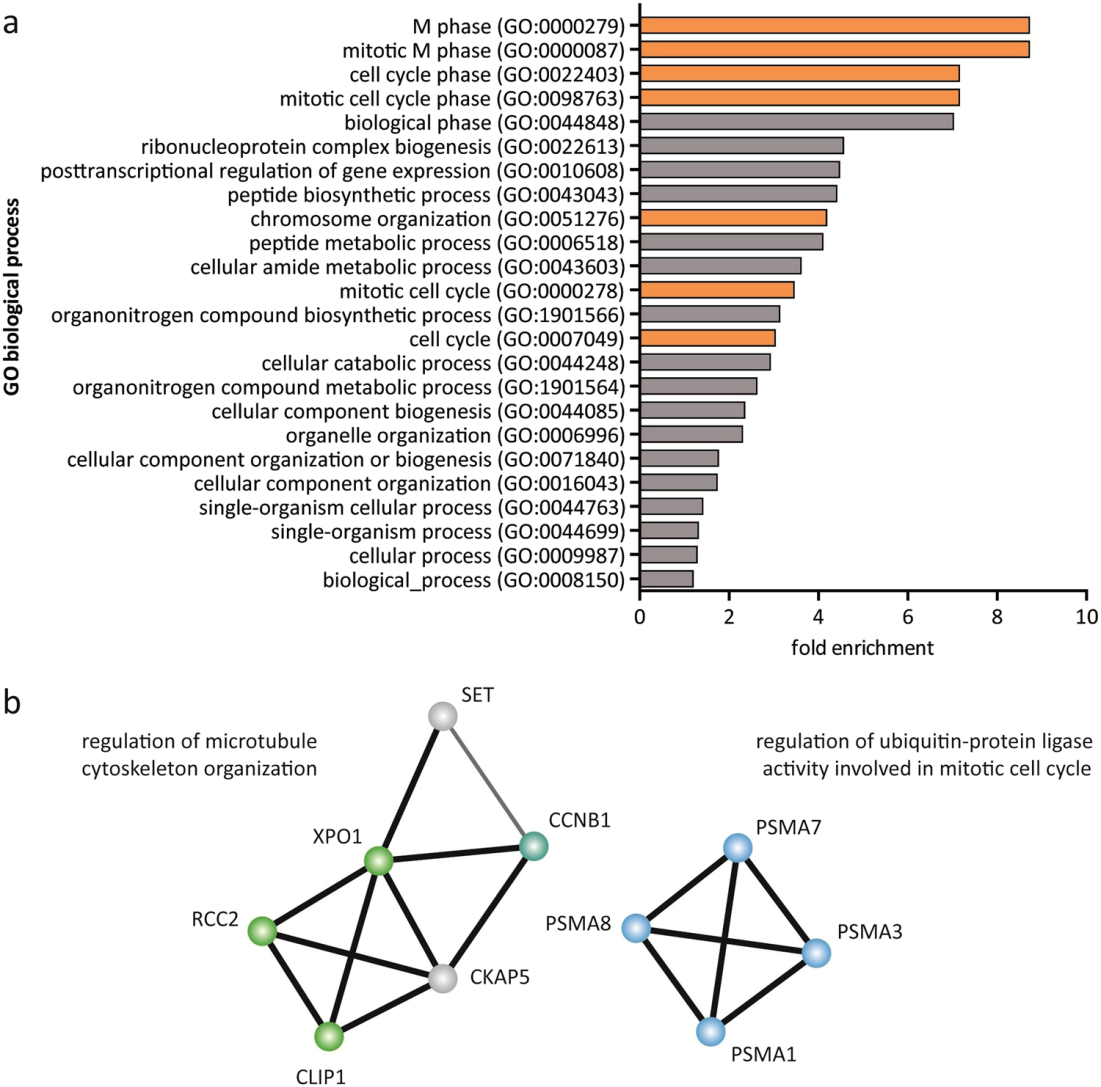
Proteins pulled down with *LINC00152* were significantly associated with M phase. (**a**) Enrichment analysis identified gene ontology (GO) terms based on biological processes that were significantly over-represented within proteins detected in *LINC00152* pulldowns. Mitosis and cell cycle-related GO terms are highlighted in orange. (**b**) String database revealed interaction networks between the proteins associated with M phase by GO analysis and their functional enrichment for microtubule cytoskeleton organization (green nodes) and ubiquitin-protein ligase activity (blue nodes) with cyclin B (CCNB1) being involved in both (cyan). Line thickness indicates the strength of protein interaction by data support.

To investigate whether the enriched proteins were involved in a specific biological process, we performed a gene ontology (GO) enrichment analysis^49^. Notably, these proteins were significantly associated with several biological processes with M phase, cell cycle and chromosome organization at the top of the list (Fig. 6a). The STRING database^50^ was used to depict known and predicted protein-protein interactions of the proteins linked to M phase by GO analysis of the *LINC00152* interactome. Interestingly, this analysis revealed two protein networks, which function in microtubule cytoskeleton organization and ubiquitin-protein ligase activity (Fig. 6b). The organization of the microtuble cytoskeleton is important for the proper attachment of the spindle microtubules to the kinetochores on sister chromatids^51^, whereas the proteasome degrades cyclin B and securin after ubiquitylation by the anaphase-promoting complex (APC/C, also known as the cyclosome)^52^. Hence, we identifed a network of proteins that is well-known to be crucial for the activity of mitotic progression pulled down with *LINC00152*.

## Discussion

The generation of loss-of-function phenotypes by RNAi provides a valuable approach to gain insights into the biological functions of lncRNAs^17, 18, 20, 22^. Therefore, our custom siRNA library targeting 638 lncRNAs upregulated in cancer provides a useful tool, which identified lncRNAs that affect cell division, including *LINC00152*. Notably, the time point of the phenotype penetration after *LINC00152* knockdown occurred relatively late during the screen and was only identified by manual scoring whereas automated analysis was not able to detect the prometaphase arrest. Therefore, it is crucial to carefully analyse and validate siRNA screens to ensure accurate and qualitative results. Using a combined bioinformatical and manual analysis approach, approximately 90% of the positive siRNA controls were found to display defects in cell proliferation demonstrating that the screen delivered meaningful results. Moreover, we identified *TERC*, which was previously reported to induce a rapid cellular growth arrest upon inhibited expression^24^ and therefore further proves the validity of the screen. Recently, a CRISPRi-based screen in seven diverse cell lines identified 499 lncRNAs required for robust cellular growth^53^. For TERC and AK096335, one single guide RNA (sgRNA) provoked a phenotype exclusively in induced pluripotent stem cells (iPSCs), but none of our 26 validated lncRNA hits were identified with this approach. Notably, the overlap between our library and this published one was small and most sgRNAs were not tested in HeLa cells.

Initially, the *LINC00152* genomic region was detected as differentially hypomethylated during hepatocarcinogenesis^54^. DNA hypomethylation is usually associated with active gene transcription^55^, which nicely fits the observation of our microarray data showing that *LINC00152* expression was significantly upregulated in lung, liver and breast cancer. Furthermore, other studies also reported elevated *LINC00152* expression in many tumours such as gastric cancer^37, 39, 40, 56–59^, hepatocellular carcinoma^38, 60^, pancreatic cancer^61^, clear cell renal cell carcinoma^62^, colorectal cancer^42, 63^ breast cancer^41^ and infantile hemangioma^64^.

Importantly, none of these reports have taken into account that *LINC00152* has an actively expressed paralog annotated as MIR4435-2HG. During our studies, we used reagents including primers, siPOOLs and raPOOLs targeting both transcripts. This is inevitable given that both genes are almost identical with only 13 mismatches in the entire transcript. Hence, previous studies need to be re-evaluated since all primers, probes and reagents will have recognized and targeted both genes. Thus, it remains to be determined whether *LINC00152* and *MIR4435-2HG* fulfil the same or different functional roles. Since both isoforms are highly similar including their up- and downstream genomic sequences, it will be technically challenging to create paralog-specific knockdowns or knockouts.

In this study, we showed that depletion of *LINC00152* in HeLa cells caused a prometaphase arrest that in some instances lasted more than ten hours. This represents a severe phenotype considering that the normal duration of mitosis is approximately one hour^65^. As a consequence, affected HeLa cells either died within mitosis, exited mitosis or displayed cytokinesis failure. We could not verify the mitotic arrest under the conditions tested in other human cell lines derived from different tissue origin (NCI-H460, HEK293, U2OS and hTERT-RPE1). This is not unexpected since also cell behaviour in response to distinct classes of antimitotic drugs is very heterogeneous and varies not only between different cell lines but even within the same cell line^66^. Moreover, the time point for detecting the prometaphase arrest is extremely crucial (compare Fig. 4b) and could have been missed easily. The investigated cell lines might have merely a mitotic delay or directly undergo apoptosis or senescence. Indeed, those cells clearly show defects in cell proliferation as evidenced by the cell viability assay. The lower cell viability rate might be attributed to an earlier or stronger reaction of these cells potentially due to a stronger checkpoint response. Thus, these experiments indicate that *LINC00152* is essential for cell cycle progression in a variety of cell lines. Overexpression of *LINC00152* did not significantly affect cell proliferation, which has been previously documented for other cell cycle regulators^7, 67^ as well. Rapidly cycling HeLa cells possibly express all factors required for fast cell cycle progression already at sufficient levels.

A number of reports confirm that *LINC00152* knockdown induces a cell cycle arrest^37, 40, 41, 62^. Thus, these studies are in agreement with our finding that *LINC00152* is essential for normal proliferation and cell cycle progression. However, we discovered for the first time that *LINC00152* is linked to an arrest specifically in mitosis. Furthermore, we find a network of proteins functioning in M phase of the cell cycle that associate with *LINC00152*. In particular, these proteins regulate the microtubule cytoskeleton organization and the regulation of ubiquitin-protein ligase activity, which are both important processes in mitosis. Additionally, the depletion of RCC2 (also known as TD-60) - one of the identified putative interaction partner of *LINC00152* - caused a strong prometaphase arrest in HeLa cells during recovery from 16 h of nocodazole treatment^68^. RCC2 depletion also associated with a cell cycle arrest during interphase and decreased mitotic entry suggesting a multifunctional behaviour of this protein^69^. RCC2 could promote dynamic interactions between kinetochores and microtubules resulting in an increased mitotic index in RCC2-depleted asynchronous HeLa cells^70^. Thus, these experiments clearly demonstrate an involvement of RCC2 in cell cycle progression which fits to the prometaphase arrest phenotype upon *LINC00152* knockdown and supports the putative functional interaction between RCC2 and *LINC00152*. Nevertheless, these studies also highlight the complexity of studying proteins that control mitosis and emphasize the need for a detailed functional analysis of the full spectrum of the 132 candidate proteins which is beyond the scope of the current study. Future studies are required to identify how *LINC00152* influences the function of these protein complexes.

It has been reported that *LINC00152* activates the PI3K/AKT/mTOR pathway by various mechanisms like raising EpCAM levels through a *cis*-regulation in hepatocellular carcinoma^38^, binding to EGFR in gastric cancer^39^ or increasing ERBB4 expression through competitive binding of miR-193a-3p in colon cancer^42^. These results were extended by research in breast cancer associating *LINC00152* with the EGFR and mTOR pathways^41^. In contrast, a study in gastric cancer detected that *LINC00152* can bind and recruit the PRC2 subunit EZH2 to p15 and p21 promoters which induces their silencing via H3K27me3 modification^40^. The PI3K/AKT/mTOR pathway as well as the CDK inhibitors p21 and p15 are important regulators for cycle progression and tumorigenesis. Notably, neither EGFR nor EZH2 were among the proteins enriched in *LINC00152* pulldowns. Thus, we could not attribute the prometaphase arrest in HeLa cells conferred by *LINC00152* knockdown using an siPOOL to minimize off-target effects to any one of these previously published interaction partners^39, 40^.

Thus, it appears that individual lncRNAs can function by mechanisms that involve different molecular partners and targets in different cells or tissue entities. For example, *metastasis-associated lung adenocarcinoma transcript 1* (*MALAT1*), which was discovered as a prognostic marker for lung cancer metastasis^71^, had been linked to two alternative mechanisms of action. On the on hand, *MALAT1* was implicated in the regulation of alternative splicing of pre-mRNAs by altering the level and phosphorylation status of two serine/arginine-rich splicing factors^72^. On the other hand, *MALAT1* was reported to interact with unmethylated Polycomb protein Pc2 at nuclear speckles to promote transcription factor E2F1 SUMOylation, resulting in the activation of growth control genes^73^. In addition, *MALAT1* regulates the expression of metastasis-associated target genes which is critical for its function in lung cancer^74^. Interestingly, all three *Malat1* knockout mouse models were reported to lack an obvious phenotype under normal physiological conditions^75–77^. Hence, large amounts of data have accumulated that link *MALAT1* to different cancer types or diseases and provided insights into its biogenesis, interaction partners and biological as well as pathophysiological roles^78^. However, the inconsistencies of the different studies demonstrate that *MALAT1* is a paradigm for the obstacles in elucidating the cellular functions and molecular mechanisms of lncRNAs. Thus, *LINC00152* may also employ different mechanisms in different cancer types and cell lines but consistently acts in an oncogenic fashion crucial for cell cycle progression. The validation and functional characterization of individual *LINC00152*-interacting proteins or complexes thereof will be an essential future task. The interaction of *LINC00152* with protein networks functioning in microtubule cytoskeleton organization and ubiquitin-protein ligase activity is an interesting starting point for future studies.

## Methods

### RNAi screen and data analysis

The siRNA screen was performed using a custom-designed library targeting 638 putative oncogenic lncRNAs (Invitrogen). The library consisted of up to five individual Silencer Select siRNAs for each target gene with more than 3100 siRNAs in total. Each plate of this library included duplicates of three positive siRNA controls targeting COPB2, KIF11 and PLK1 protein-coding RNAs, which are known to affect cell viability as well as duplicates of three non‐targeting negative siRNA controls named NC A, B and D (Invitrogen). Additionally, the library also contained control siRNAs for mitotic defects targeting three proteins (CENPA, ZW10, HJURP) and six centromeric satellite RNAs putatively involved in mitosis. HeLa cells (850 per well) were transfected with siRNA (30 nM) using DharmaFECT1 reagent (GE Healthcare). Cells were imaged starting at 4 hours post‐transfection up to 72 hours on an inverted wide-field Olympus Biosystems IX81 microscope in an incubator chamber. Images were acquired at 10x magnification with the Scan^R software every 30 min in a GFP and TexasRed channel. The image data was analysed by automatic segmentation and tracking of the cell nuclei^79^ to calculate the MitosisCount. This value represented all mitotic events that occurred per image over all time points counted when one track split into two tracks. The results obtained from the automatic image analysis of the primary screen were used to calculate robust z-scores normalized to the median value of the sample wells by the open source R package cellHTS2^80^. We defined hits if at least two siRNAs were above the z-score cut-off of 2. In addition, the data was analysed manually for any kind of aberrant phenotype by assigning a manual score to the last time point ranging from 1 (normal) to 5 (strong phenotype). In this analysis, a lncRNA was considered as hit when targeted by at least three siRNAs with a manual score ≥3 or at least two siRNAs with a manual score ≥4.

To validate the siRNA screening results, all siRNAs for every hit were redistributed on a new plate and the RNAi experiment was repeated. However, in this case, it was not suitable to calculate the z-score since it involved the plate median. Therefore, the values of the MitosisCount were divided by the median of the negative controls (raw/mnc). In this analysis, a validated lncRNA hit was defined when the same two or more siRNAs, that had an effect in the screen, showed a raw/mnc ≤ 0.8 or a manual score ≥3.

### siPOOL knockdown

For *LINC00152* knockdown using siPOOLs^32^, cells were reverse transfected with 30 nM (final concentration) of siControl or siLINC00152 (siTOOLs Biotech) using 2 μl DharmaFECT1 reagent (GE Healthcare) in 6-well plates. Cells were lysed in 1 ml TRI reagent for RNA extraction or 1x RIPA buffer for protein extraction 60 hours post transfection. siPOOL sequences are listed in Supplementary Table S4.

### Western blot

Protein samples were prepared with RIPA buffer. Therefore, cells were washed with ice-cold phosphate buffered saline (PBS), scraped from the culture dish and lysed for 10 min on ice in 100 μl RIPA buffer per well of a 6-well plate. Subsequently, crude lysates were centrifuged for 15 min at 17,000 g. The supernatant was transferred to a fresh tube. Protein concentration was determined with a Bicinchoninic Acid (BCA) assay and samples were separated by sodium dodecyl sulphate polyacrylamide gel electrophoresis, using 12% self-cast gels (Biorad). After separation, the samples were transferred from the gel to a methanol-activated PVDF membrane (Roche) at 90 V for 2.5 hours at 4 °C using a wet blotting system (Biorad). The following primary antibodies were used according to the manufacturer’s recommendations: anti-phospho-Lamin A/C (1:1000 in TBS-T + 5% milk, #13448 Cell Signaling) and anti-beta-Actin (1:20,000 in TBS-T + 5% milk, A2228 Sigma-Aldrich). Detection was performed with HRP-coupled secondary antibodies.

### CellTiter-Glo luminescent cell viability assay

Cell viability was analysed with the CellTiter-Glo Luminescent Cell Viability Assay (Promega, 1:4 diluted in PBS) according to the manufacturer’s recommendation.

### RNA pulldown, SP3 cleanup and mass spectrometry analysis

Comprehensive identification of RNA binding proteins by mass spectrometry (ChIRP-MS) was performed according to previous studies^45, 81^ with the following modifications. For each sample, 40 million HeLa cells were crosslinked with 3% of formaldehyde and sonicated in 1 ml of cell lysis buffer (50 mM Tris pH 7.0, 10 mM EDTA, 1% SDS) at 4 °C at the highest setting with 30 seconds on and 45 seconds off pulse intervals for 280 cycles in a Bioruptor (Diagenode). Sonicated and precleared lysates were incubated with 2 ml of hybridization buffer (750 mM NaCl, 50 mM Tris pH 7.0, 1 mM EDTA pH 8.0, 1% SDS, 15% formamide) and 200 pmol of biotinylated raPOOL (siTOOLs Biotech) probes at 37 °C for 4 hours with shaking. Next, 2 ml of C-1 magnetic beads (Invitrogen) were added to the hybridized tubes and incubated for 30 min at 37 °C with shaking. After washing of the beads, the captured proteins were eluted twice with 500 µl of biotin elution buffer (12.5 mM biotin [Invitrogen], 7.5 mM HEPES pH 7.5, 75 mM NaCl, 1.5 mM EDTA, 0.15% N-lauroylsarcosine, 0.02% sodium deoxycholate) at 28 °C for 20 min and 65 °C for 10 min. raPOOL sequences are listed in Supplementary Table S5.

Protein cleanup was performed as described with the following modifications^82^. Samples were decrosslinked over night at 65 °C and RNA was digested with benzonase for 1 hour at 37 °C. Iodoacetamide was added to a concentration of 12.5 M and allowed to react for 20 min in the dark before adding formic acid (Biosolve) to a final concentration of 0.5%. Next, samples were supplemented with 5 µl of a 1:1 mixture of hydrophobic and hydrophilic Sera-Mag magnetic carboxylate modified beads (Sigma-Aldrich) and mixed. One volume (1 ml) of acetonitrile was added, vigorously mixed and incubated for 30 min on a rotator. Beads were washed twice with 1 ml of 70% ethanol and once with 1 ml of acetonitrile. For overnight digestion with 0.2 µg of trypsin/lys-C, beads were dissolved in 10 ml of 50 mM tetraethylammonium bromide pH 8.0. To facilitate peptide binding, 200 µl of acetonitrile were added to the beads and the mixture was incubated for 10 min. Again, the beads were washed twice with 200 µl of acetonitrile before eluting the peptides in 10 µl of 4% DMSO by sonication. Finally, the eluent was acidified with formic acid to 0.1% final concentration.

LC-MS analysis was performed on an Easy-nLC system connected to a QExactive HF mass spectrometer. For peptide and protein identification as well as quantification, the MaxQuant software package was employed^83^. iBAQ scores were used as approximate measures for the absolute protein amounts calculated as the sum of all peptide peak intensities divided by the number of theoretically observable tryptic peptides^84^.

## Electronic supplementary material

Supplementary Figures & Methods

Supplementary Tables
