# Supplementary material for: The long non-coding RNA LINC00152 is essential for cell cycle progression through mitosis in HeLa cells
Source: Sci Rep. 2017 May 23;7:2265. doi: 10.1038/s41598-017-02357-0 (PMC5442156; doi:10.1038/s41598-017-02357-0)
Supplement: Supplementary file 1 — Supplementary Figures & Methods [file 41598_2017_2357_MOESM1_ESM.pdf]

The long non-coding RNA *LINC00152* is essential for cell cycle progression through mitosis in HeLa cells

Linda Nötzold[1,2,3], Lukas Frank[1], Minakshi Gandhi[1], Maria Polycarpou-Schwarz[1], Matthias Groß[1], Manuel Gunkel[4], Nina Beil[4], Holger Erfle[4], Nathalie Harder[5,14], Karl Rohr[5], Jakob Trendel[6,7], Jeroen Krijgsveld[6,7], Thomas Longerich[8,9], Peter Schirmacher[9], Michael Boutros[10], Sylvia Erhardt[2,3], Sven Diederichs[1,3,9,11,12,13]

SUPPLEMENTS

Supplementary Figures & Legends

Supplementary Methods

Supplementary References

Supplementary Table Legends

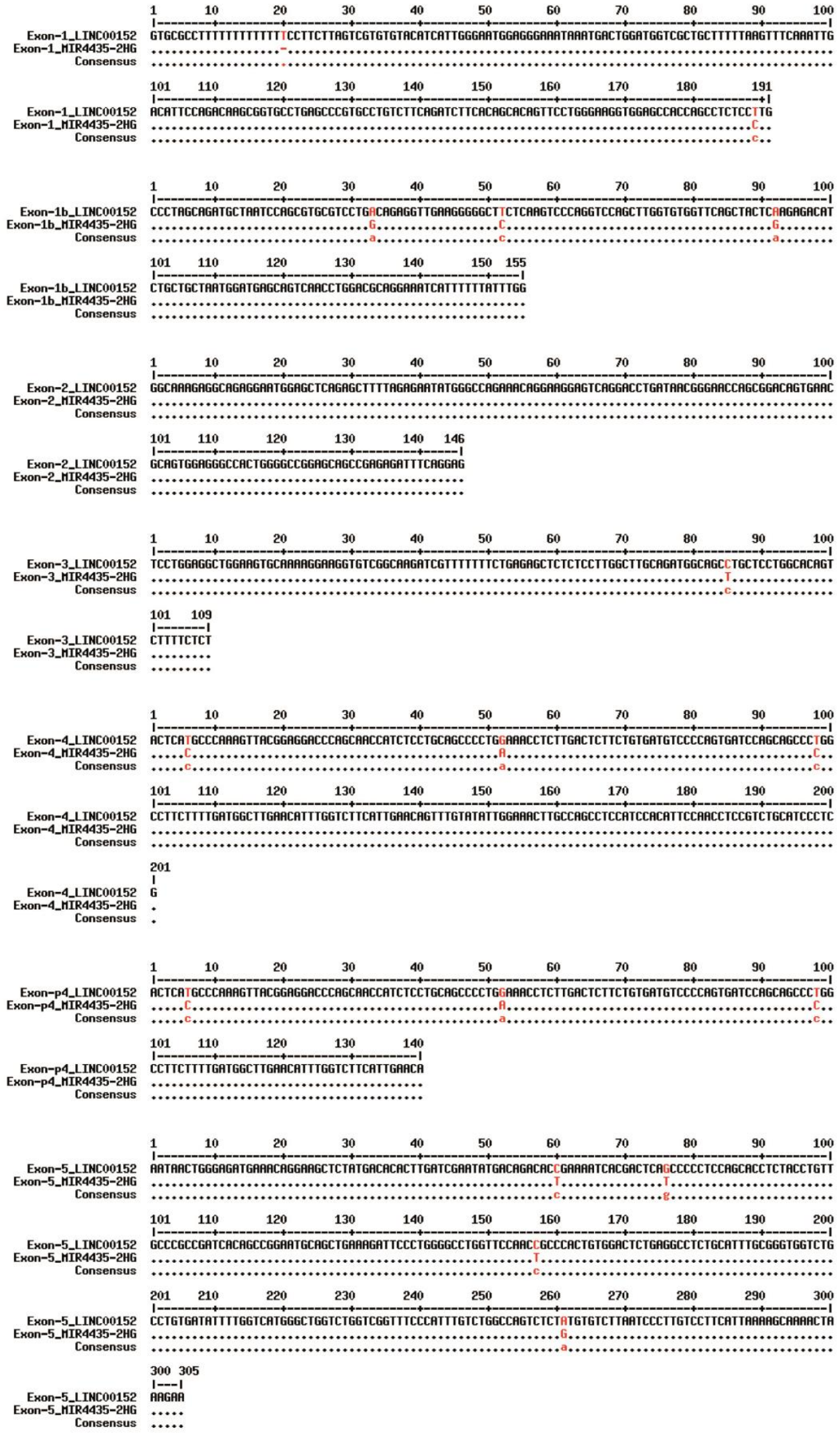

Supplementary Figure S1: Sequence alignment of *LINC00152* and *MIR4435-2HG*. All exons were aligned separately with mismatches highlighted in red by Multalin[1].

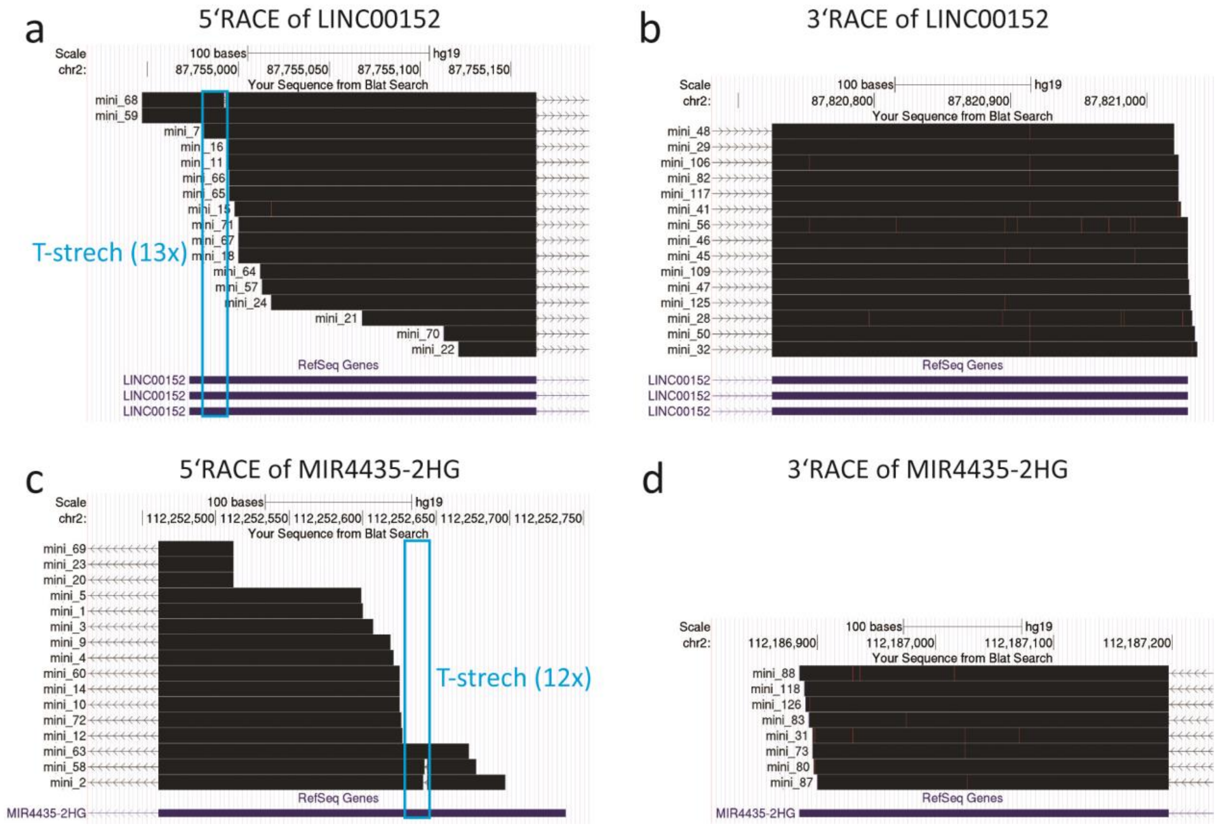

Supplementary Figure S2: RACE analysis confirmed annotated 5’ and 3’ ends of *LINC00152* and *MIR4435-2HG*. Cloned RACE (rapid amplification of cDNA ends) fragments were sequenced and aligned in the UCSC Human Genome Browser hg19 assembly with (**a**, **b**) *LINC00152* and (**c**, **d**) *MIR4435-2HG* to identify the 5’ and 3’ ends of both transcripts. Blue box indicates repeats of thymidine (T-stretch).

a

Transcript	Longest ORF (aa)
LINC00152 ex15	52[a)]
LINC00152 ex145	70[b)]
MIR4435-2HG ex15	52[a)]
MIR4435-2HG ex145	52[a)]

a) MTDTENHDSAPSSTSTCCPPITAGMQLKDSLGPGSNRPLWTLRPLHLRVVCL*
b) MPKVTEDPATISCSPWKPLDSSVMSPVIQQPWPSFDGLNIWSSLNSLYIGNLPASIHIPTSVCIPRITGR*

b

ID	C/NC	CODING POTENTIAL SCORE
MALAT1_(NR_002819.3)	noncoding	-1.00788
NEAT1_(NR_131012.1)	noncoding (weak)	-0.835983
PPIA_(NM_021130.4)	coding (weak)	0.182016
GAPDH_(NM_002046.5)	coding	2.27512

c

ID	C/NC	CODING POTENTIAL SCORE
LINC00152ex15	noncoding (weak)	-0.878362
LINC00152ex145	noncoding (weak)	-0.81983
MIR4435-2HGex15	noncoding (weak)	-0.813744
MIR4435-2HGex145	noncoding (weak)	-0.856831

d

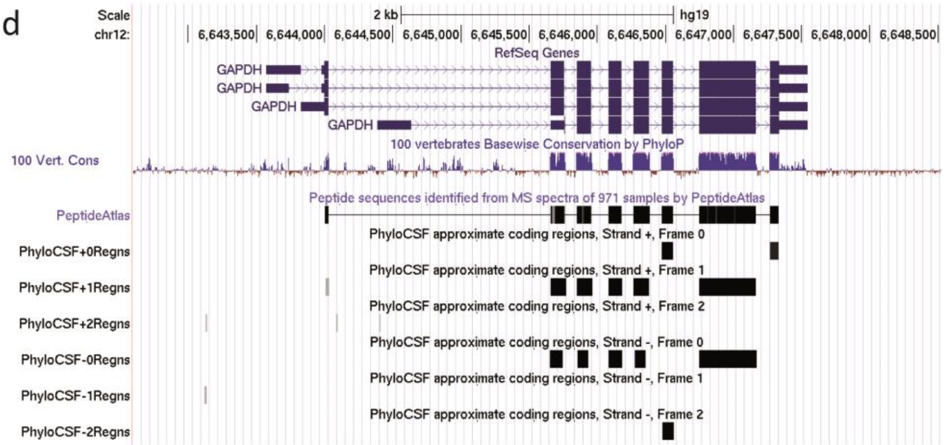

e

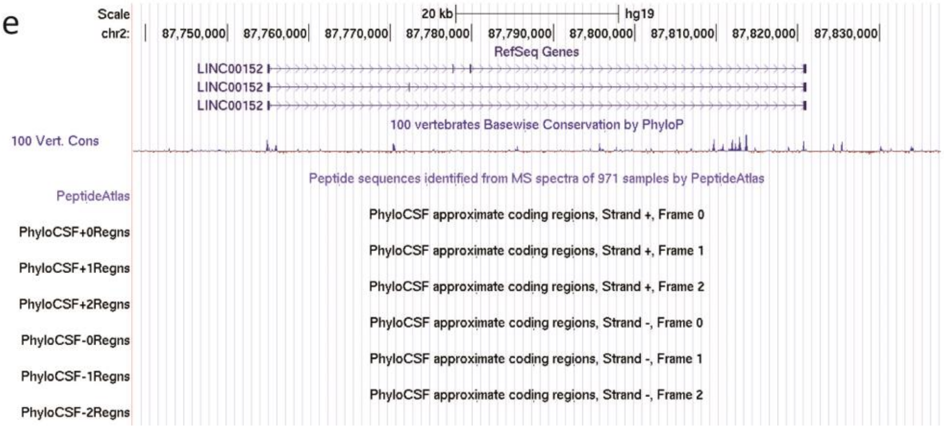

Supplementary Figure S3: ***In silico*** **analysis indicated non-coding properties of** ***LINC00152*****.** (**a**) Longest full open reading frames (ORFs) of *LINC00152* and *MIR4435-2HG* splice variants ex15 and ex145. (**b**) The coding potential scores (CPSs) for two protein-coding genes (GAPDH and Cyclophilin A (PPIA)) and two lncRNAs (MALAT1 and NEAT1) were calculated by the Coding Potential Calculator. (**c**) The CPSs for splice variants ex15 and ex145 of both *LINC00152* and *MIR4435-2HG*. (**d**, **e**) UCSC tracks for Vertebrate Conservation, PeptideAtlas and PhyloCSF Regions for each of the six frames at the (**d**) GAPDH and the (**e**) *LINC00152* locus.

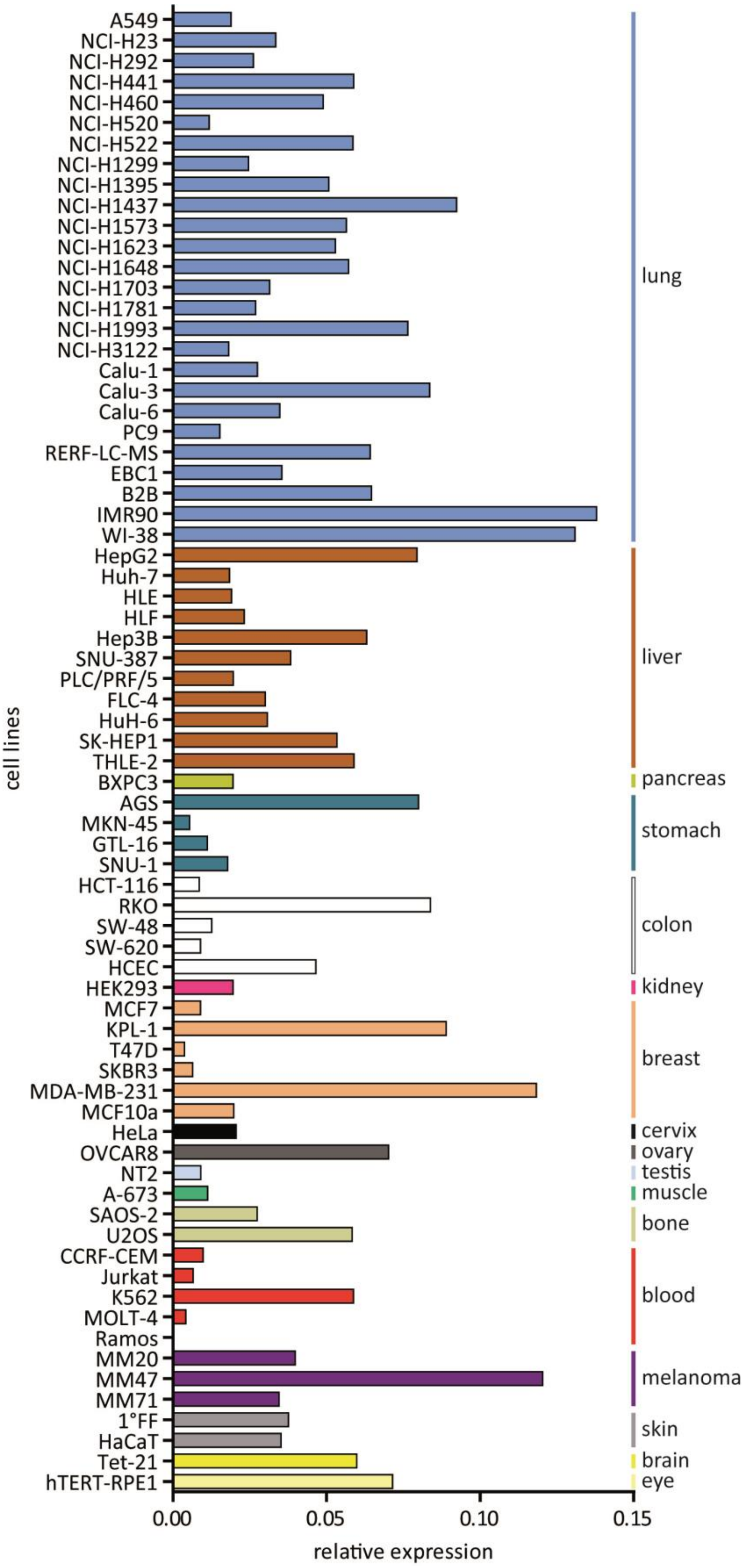

Supplementary Figure S4: *LINC00152* expression in variety of human cell lines from different tissue origin. Relative expression of *LINC00152* in a set of 72 cell lines (left) from different tissue origin (right) normalized to Cyclophilin A expression (N=1 with technical duplicates).

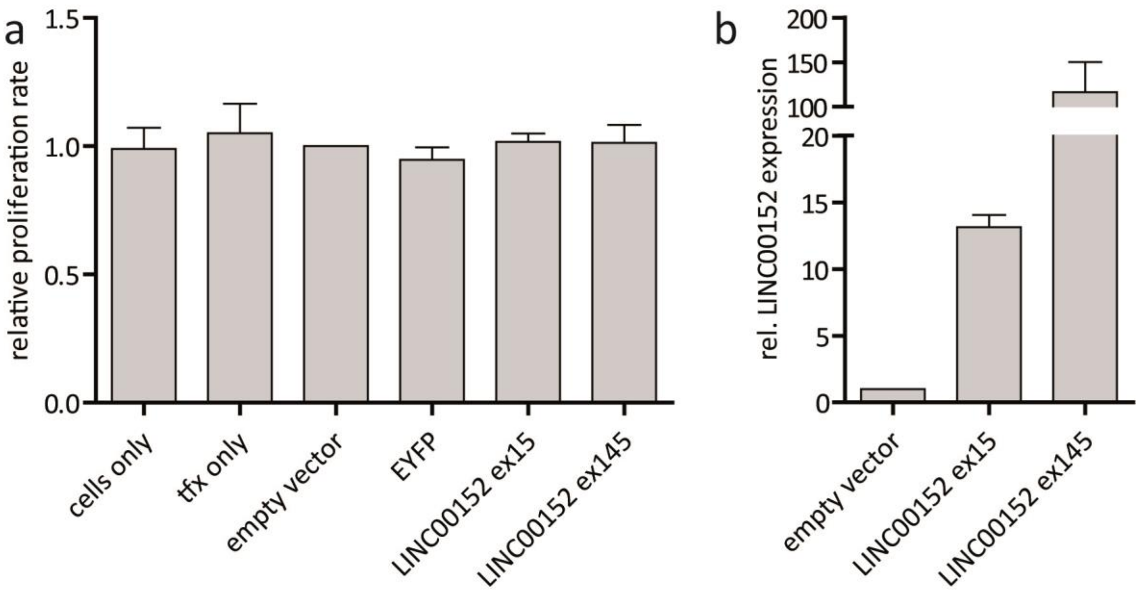

Supplementary Figure S5: Analysis of cell proliferation after *LINC00152* overexpression. (**a**) HeLa cells were transfected with *LINC00152* splice variant ex15 or ex145 overexpression vectors. Cell proliferation was measured 72 h after transfection and normalized to the empty vector control. (**b**) Overexpression of *LINC00152* splice variant ex15 and ex145 was assessed by qRT-PCR and normalized to endogenous *LINC00152* expression in the empty vector control. N=3. Error bars indicate SEM.

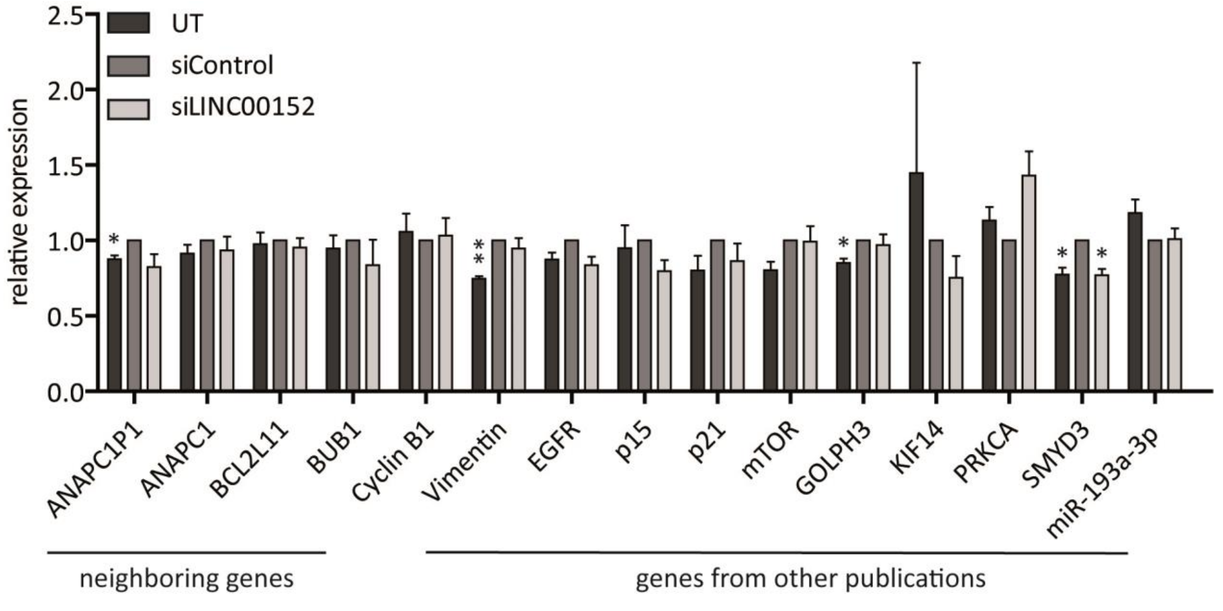

Supplementary Figure S6: Impact of *LINC00152* depletion on the expression of associated genes. Relative expression levels of *LINC00152*-neighboring genes, mitotic cyclin B1 and genes reported in other publications to be deregulated upon *LINC00152* knockdown were determined by RT-qPCR. Of note, EpCAM, E-Cadherin and ERBB4 were not detected in HeLa cells. UT = untreated; N≥3. Error bars indicate SEM.* $p<0.05$; ** $p<0.01$; *** $p<0.001$.

Supplementary Methods

Cell culture

HeLa and HEK293 cells were cultivated in Dulbecco's modified Eagle's medium (DMEM; Sigma-Aldrich) supplemented with 10% Fetal Calf Serum (FCS) (Thermo Fisher Scientific) and 1% L-glutamine (Sigma-Aldrich). NCI-H460 cells were cultivated in RPMI-1640 medium (Sigma-Aldrich) supplemented with 10% FCS. U2OS cells were cultivated in McCoy's 5A medium (Sigma-Aldrich) supplemented with 10% FCS and 1% L-glutamine. hTERT-RPE-1 cells were cultivated in DMEM:F12 medium (Life Technologies) supplemented with 10% FCS. All cell lines were cultured at 37°C in a 5% CO_2 atmosphere.

RNA extraction, reverse transcription and qPCR

RNA was isolated from TRI Reagent (Sigma-Aldrich) lysates. Isolation was performed according to the manufacturer's recommendation. Synthesis of cDNA from 1 µg RNA was performed with Random Hexamer Primers and RevertAid Reverse Transcriptase (both Thermo Fisher Scientific). Expression of target genes was detected with qPCR using specific primer pairs and the Power SYBR Green Master Mix (Thermo Fisher Scientific). Generally, relative expression was determined using the $\Delta\Delta C_T$ method. All values were normalized to Cyclophilin A and compared to a respective control sample. Primers are listed in Supplementary Table S6.

Cellular fractionation

Cytoplasmic, nucleoplasmic and chromatin fractions for RNA extraction were prepared according to a previously published protocol[2].

Statistical analysis

F-test was performed to test homoscedasticity meaning if two sample groups have the same variance. In case of equal variance (F-test $p>0.05$), Student’s t-test was performed. Otherwise ($p\leq0.05$), Welch’s t-test was performed. All statistical analyses were defined as significant for p-values <0.05.

RACE

For performing both 5′- and 3′-rapid amplification of cDNA ends (RACE), the SMARTer RACE cDNA Amplification Kit (Clontech) was used and all reactions were performed according to the manufacturer's protocol. All gene-specific primers (GSPs) and nested GSPs (NGSPs) are listed in Supplementary Table S7.

Plasmids

Cloning of *LINC00152* splice variants ex15 and ex145 into the pEF-DEST51 expression vector was performed using the Gateway system (Invitrogen). For creating the entry clones, *attB*-flanked PCR products were generated and recombined individually into the *attP*-containing donor vector pDONR221 through a BP clonase reaction. Entry clone plasmids were further recombined in an LR clonase reaction with the *attR*-containing destination vector pEF-DEST51 to obtain expression clones that carry the *LINC00152* splice variants or EYFP under the control of an EF1-α promoter. Primers are listed in Supplementary Table S7.

Proliferation assay

Cells were seeded in black 96-well plates with clear bottoms and after 24 hours transfected with 100 ng plasmid DNA using Polyetherimid (PEI). Cell proliferation was analyzed 72 hours after transfection with the Cell Proliferation ELISA BrdU assay (Roche) according to the manufacturer's recommendations. Briefly, cells were labeled with Bromodeoxyuridine (BrdU) by incubating the cells with the BrdU solution at 37°C for 6 hours. The chemiluminescence was measured with a Fluoroskan Ascent FL luminometer (Thermo Scientific).

Supplementary References

1 Corpet, F. Multiple sequence alignment with hierarchical clustering. *Nucleic Acids Res* **16**, 10881-10890 (1988).

2 Gagnon, K. T., Li, L., Janowski, B. A. & Corey, D. R. Analysis of nuclear RNA interference in human cells by subcellular fractionation and Argonaute loading. *Nat Protoc* **9**, 2045-2060, doi:10.1038/nprot.2014.135 (2014).

Supplementary Table Legends

Supplementary Table S1: Bioinformatical and manual analysis of the time-lapse siRNA screens.

Supplementary Table S2: Summary of putative hits from the primary screen and secondary validation.

Supplementary Table S3: iBAQ scores of proteins enriched in *LINC00152* pulldowns after biotin elution.

Supplementary Table S4: Sequences of siPOOLs used as negative control and siPOOLs targeting *LINC00152*.

Supplementary Table S5: Sequences of *LINC00152* and *lacZ* raPOOLs used for RAP.

Supplementary Table S6: Primers used for RT-qPCR.

Supplementary Table S7: Sequences of gene-specific primers (GSPs) and nested GSPs (NGSPs) for *LINC00152* used for RACE.
